# Supplementary material for: Rift Valley fever virus and Coxiella burnetii infections among febrile patients, Eastern Ethiopia
Source: PLoS Negl Trop Dis. 2025 Aug 13;19(8):e0013375. doi: 10.1371/journal.pntd.0013375 (PMC12364338; doi:10.1371/journal.pntd.0013375)
Supplement: Attachment — Submitted filename: Response to Reviewers.docx [file pntd.0013375.s002.docx]

**A point-by-point response to reviewer comments**

**Notes on major changes:**

- In the previous version:
- We screened serum samples for IgM and IgG antibodies simultaneously for the RVF virus but reported as we did first IgG antibodies and then IgM which is incorrect and confusing. We have revised this accordingly.
- In this version:
- We re-analyzed all blood samples for viral RNA and *C. burnetii* bacterial DNA to detect genomes that may have been present before the elicits of IgM (viremia or bacteremia but IgM-negative), while also resolving methodological ambiguities observed in the previous version.
- Clearly distinguished between acute (detection of IgM antibodies in case of RVF and anti-Phase II antibodies in case of *C. burnetii*)
- All line numbers cited in this document correspond to the revised version of the manuscript.

**Reviewers' Comments:**

**Reviewer #1: Abstract**

1) The way lines 20-21 are written: I think it should be IgM-positive samples that should be sequenced as they are linked to an acute infection, whereas IgG samples should be associated with chronic or permanent infection…Can authors clarify this? Also, the whole sentence does not make sense, because it is inaccurate “…IgG-positive samples were tested for genomic markers using real-time polymerase chain reaction”.

**Author’s response:**

We thank the reviewer for this valuable observation. We agree that only IgM-positive samples should be selected for PCR testing. The mention of IgG was a typographical error, as PCR was performed exclusively on acute-phase markers (IgM). In the revised manuscript, we have:

- Retested all samples for viral RNA and bacterial DNA to clear these ambiguities
- Clearly distinguished between acute (IgM) and past (IgG) infections and revised the Methods section to explicitly state this approach.

We have revised the text (Lines 20-21).

2) Can authors explain the meaning of this sentence? It looks confusing to me: "IgG-positive samples were tested for genomic markers using real-time polymerase chain reaction".

**Author's response**:

We thank the reviewer for this comment. We have rephrased the text (Lines 20-21).

3) Conclusion to be rewritten.

**Author’s response**:

We thank the reviewer for this suggestion. We have revised the Conclusion (Lines 32-36).

**Introduction**

4) Use active form as much as possible while maintaining a classical structure of a sentence (Subject + Verb + Object) throughout the manuscript. For example, lines 108-100, 112-113, etc.

**Author’s response**:

We thank the reviewer for this comment. We have revised the text to use active voice where appropriate (Lines 109–111). However, we retained passive voice or past tense where active construction would require first-person pronouns (we, I), as these are generally discouraged in manuscript writing. We have also carefully checked all tense usage in the revised manuscript.

**Methods**

5) Since all symptoms were self-reported could authors include or address any bias related to that?

**Author’s response**:

We thank the reviewer for raising this important methodological concern. We acknowledge that self-reported symptoms introduce bias, which could affect the reported results. Although this limitation is inherent to questionnaire-based studies, we tried reducing it by:

- Limiting the recall time to less than 30 days (Line 271).
- Cross-validating symptoms reports with clinical records (311-314).

We described this as a limitation (393-395).

6) Lines 116-119: rephrase. Can the authors clarify how they objectivized these elements? "Known infection", "clinical evidence of a specific cause of fever", "positivity to Malaria"?

**Author’s response**:

We thank the reviewer for requesting clarification on how we defined these terms. We defined all terms using our integrated clinical-laboratory assessment protocol, including:

1. Clinical evaluation:

- Trained health professionals conducted examinations of the patient for routine clinical workups:
- Detailed history-taking (including HIV, Tb, recent surgery, or any chronic conditions)
- Physical examination, and vital sign documentation (Lines 119-121; 141-143)

2. Laboratory testing:

- Prescribe laboratory tests such as rapid Syphilis, HIV, HBsAg, etc.,
- Malaria positivity: Confirmed by blood film microscopy (Lines 162–164), excluding malaria-positive febrile patients (Lines 120-121).

We used this dual approach to operationally define the terms.

7) Sample size estimation: Instead of putting the raw formula, just mention the name of the formula and the reference.

**Author’s response**:

We appreciate this helpful suggestion. We have revised the manuscript by excluding the formula while citing its original reference (Line 124).

8) On which basis the prevalence of RVF Kenya was chosen, why not from Egypt or Tanzania? Can authors lay more on that?

**Author’s response**:

We appreciate this thoughtful question. Kenya was selected primarily on the basis:

- **Geographic/ ecological relevance**: Ethiopia and Kenya share extensive borders and similar ecological conditions that favor RVF transmission (e.g., pastoralist communities, flood-prone Rift Valley ecosystems).
- **Epidemiological patterns**: Kenya experiences recurrent outbreaks (unlike Ethiopia), providing relevant transmission data for risk estimation.
- **Shared regional spillover risk**: Cross-border human, livestock, and vector movement increases shared exposure risk.

We thought using prevalence data from Kenya was more relevant and scientifically justifiable for calculating our sample size (Sumaye *et al.*, 2019) because Ethiopia has: (1) distinct climatic and environmental differences (e.g., Egypt's Nile Delta ecosystem), (2) the absence of direct border contact between with these countries, and (3) absence of share cultural and risk patterns etc.

9) Lines 127-128: rephrase this sentence or check the punctuation. What do the authors mean by the lottery method? Is there any reference for that, or can the authors explain?

**Author’s response**:

We appreciate the reviewer's attention to methodological clarity. We have:

- Rephrased the sentence (Lines 131)
- Added detailed methodological explanations of the lottery-based randomization process for health center selection (Lines 131-133).

10) Lines 130-132: the recruitment method is blurred, not sure it's clear for the reader. If fever was the main inclusion criterion, it means that only febrile patients were randomly selected, not all patients. In that case, the sentence should read as follows "Febrile participants were randomly recruited using a predetermined interval of 2 or 3 among them or febrile patients…". Otherwise, the way it is written, the interval of 2 or 3 corresponds to all patients, not febrile ones. Authors can clarify.

**Author’s response**:

We thank the reviewer for this insight and for requesting clarification regarding the recruitment process. Yes, fever is indeed our primary inclusion criterion, and only febrile patients were included in the random selection process. We have revised the text (Lines 136-137).

11) Lines 133-134: The authors can explain shortly the procedure. Were authors contacting febrile or all patients?

**Author’s response**:

We thank the reviewer for this thoughtful observation. We enrolled febrile patients with fever who met inclusion criteria (Lines 116–117) and have revised the text (Lines 137–138).

12) Line 140: specify how the pretest was carried out

**Author’s response**:

We thank the reviewer for this valuable input. Before the implementation of the study, we pretested procedures (including questionnaires, interview guides, and laboratory protocols) on 5% of febrile patients (n=21) at two non-study health facilities. This pretest identified and enabled the correction of several issues (Lines 144–148), including:

- Unclear questionnaire items
- Interview timing inconsistencies, and
- Logistical challenges in sample transport.

13) Lines 141-143: not sure that tube as per the international codification…Check or provide the reference.

**Author’s response**:

We thank the reviewer for this important observation. We have included the complete product specifications with international codification standards (Lines 150-151).

14) Lines 147-148: drop, not useful

**Author’s response**:

We thank the reviewer for this suggestion. We agree that this content does not contribute to the preciseness of the manuscript and have removed it.

15) Lines 144-145: real-time polymerase chain reaction corresponds to rt PCR, not RT-PCR which is Retro-Transcriptase polymerase chain reaction. Authors should choose what they used for the study. May I recall that we have here an RNA virus and a DNA organism (bacterium).

**Author’s response**:

We thank the reviewer for this important technical clarification. We confirm that:

- For RVF RNA virus: We used Reverse Transcription PCR (RT-PCR), and
- For *C. burnetii* DNA: We used real-time PCR

We have rephrased the text (Lines 152-153) and consistently use these appropriate terms.

16) Lines 145-146, if authors talk about centrifugation, it is better for them to also mention the centrifuge brand.

**Author’s response**:

We appreciate this valuable suggestion. We have included the centrifuge brand (Lines 156-157).

17) Specify the ELISA instrument brand used for the RVFV runs.

**Author’s response**:

We thank the reviewer for this important comment. For consistency, the same ELISA plate washer (Lines 178-179) and absorbance reader (Lines 182-183) were used throughout the study. We have streamlined this information in the manuscript to eliminate redundancy.

18) Authors can specify the type of ELISA used for RVF screening, as they did for Q fever screening?

**Author’s response**:

We appreciate the reviewer's careful attention to methodological details. We have specified the ELISA kits (Lines 182-183) which we used to process all samples (Lines 210-211).

19) Lines 161-162 are not clear enough for the reader. Authors can explain the added value of RVFV and dilution buffer adds-on.

**Author’s response**:

We appreciate the reviewer's valuable feedback. We have addressed this concern by including further information: to mention a few:

1. **Added the volume of dilution buffer, and its importance:** The RVF virus antigens were pre-coated by the manufacturer on ELISA plates. Following their protocol, we added the specified buffer volumes (Lines 172-173), which serve two critical functions:
   - normalizing sample viscosity to ensure consistent microplate loading,
   - minimizing non-specific binding to reduce background interference,
   - maintaining antigen-antibody binding specificity.
2. **Methodological clarifications:** We have added a brief procedure from sample preparation steps to interpretation (Lines 172-186).
20) Lines 168-170 is not clear to me: please present the kit and its targets, then the manufacturer’s performance.

**Author’s response:**

We thank the reviewer for this important suggestion. We have revised and included:

- Kit specifications and exact antibody targets (Lines 196-197), and
- Manufacturer-reported performance metrics (Lines 204-205).

21) Lines 164-171 to be rephrased.

**Author’s response:**

Thanks, we briefly included the procedure in the Methods section (Lines 195–204).

22) Lines 177-180: when samples were IgG positive, they went through IgM screening as per the authors’ algorithm (lines 154-159, 205-206): why did authors not screen only IgM positive samples for more accuracy?

**Author’s response:**

We appreciate the reviewer's insightful question. We addressed this by re-analysis of all blood samples and have revised the manuscript accordingly. In brief,

1. **For RVF virus:**

- All samples were IgM-negative (Line 285), making IgM-based screening unfeasible for detection of viral RNA. As a result, we re-analyzed all blood samples for RT-PCR (Line 213-216) to:
- Detect potential pre-seroconversion cases (viremic but IgM-negative)
- Rule out active infection in IgG-positive febrile patients

2. **PCR testing for *C. burnetii*:**

Previous studies have reported that Phase II IgG antibodies appear concurrently with IgM but persist longer (Figure X). This extended seropositivity window makes Phase II IgG-positive samples suitable candidates for real-time PCR confirmation. Nevertheless, we performed real-time PCR re-analysis on all blood samples (Lines 213-216) [1, 2] to.

- Detect cases where bacterial DNA might precede seroconversion
- Capture early-stage infections before Phase II IgG antibody elicits

(van Wijk *et al.*, 2011)

23) Lines 188-190: were the Ct-values thresholds set by the manufacturer or the authors?

**Author’s response**:

In our original submission, we inappropriately reported our laboratory's clinical reference values for pathogen detection. We recognize this approach lacked scientific justification. In the revised manuscript, we have updated the Ct values to those recommended (Lines 224-225).

24) Is Table 1 for *C. burnetii* or for both pathogens? Please specify and adjust the table’s title.

**Author’s response**:

We thank the reviewer for their valuable feedback. We have revised the table title to include both pathogens.

25) Lines 194-196: can authors specify the target product profile for all ELISA assays IDVet, Abbexa, and IDEXX to justify the way the algorithm was set? Or authors can provide some arguments on why they should start with IDvet and then go for Abbexa and IDEXX for QC. Maybe a reference can be sufficient to illustrate this. Idem for PCR kits.

**Author’s response**:

We thank the reviewer for requesting clarification/ references to support our argument. Our multi-layered approach was designed to maximize detection accuracy through sequential verification. We have included a supporting reference (Lines 166-210). Briefly,

**ELISA selection rationale**:

We select primary, secondary and supplementary screening ELISAs based on the following criteria

**Primary and secondary ELISA:**

- **IDvet ELISA**: targeting total and specific antibodies against both pathogen antigens (IgG/ IgM in RVF while for *C. burnetii* Phase I and Phase II)
- **Indirect ELISA (Virion\Serion)**: Targets *C. burnetii* Phase I/II IgG antibodies
- **High performance**: Sensitivity/specificity of all primary and secondary ELISA are 91–100% for antibody detection.

**Supplementary ELISA: These were used to complement the primary screening results**

- Abbexa RVF IgG ELISA: Targeting IgG
- IDEXX Q Fever ELISA: Targeting IgG
- They have also high-performance sensitivity/specificity ranging from 90-100%.

**PCR selection rationale** (Lines 213-226)**:**

**Primary PCR kits:**

- Primers/probes amplify conserved regions (RVFV L-segment and *C. burnetii* IS1111 element) of the respective pathogens irrespective of the source of the samples.

**PCR supplementary tests:**

All extracts were retested to exclude false positives/negatives (Lines 231-233).

- *RealStar RVFV RT-PCR Kit:
- *AmpliSens C. burnetii PCR Kit:

**NB:**

* These are commercial kits for which the manufacturer did not specify the target genes.

**Results**

26) Provide a flow diagram for lines 222-227. After this provide demographics and clinical results, thereafter, go the test results.

**Author’s response**:

We sincerely thank the reviewer for this constructive suggestion. We have added a flow chart (Fig. 2) illustrating the study design and sample screening algorithm (Lines 259-262).

27) Table 2, the title can be summarized.

**Author’s response**:

We thank the reviewer for this helpful suggestion. We have revised the title of Table 2 to be more concise while preserving its key information.

28) Line 243: is RVF positivity referred to IgM-positive samples?

**Author’s response**:

We appreciate the reviewer's attention to this point. No, our reported RVF seropositivity refers exclusively to IgG antibody detection, as no IgM-positive cases were identified. We have revised the text (Line 283).

29) If this assumption is true “None of the RVF virus IgG-positive patients tested positive for RVF virus IgM” (lines 244-245): why authors should sequence IgG positive samples?

**Author’s response**:

We thank the reviewer for this important question and acknowledge how our original text could have confused. We have revised the text (Line 285).

**To clarify key points:**

1. **Initial plan**: Our protocol proposed the detection of RNA from IgM-positive samples.
2. **Actual findings:** No sample tested IgM-positive
3. **Way forward:**

- We re-analyzed all blood samples (IgG positive and IgG negative) with RT-PCR to:
- Identify pre-seroconversion infections (viral RNA before IgM development)
- Rule out false negatives from delayed IgM antibody production

30) Lines 257-259: if the IgG positive samples selected (RVF or Coxiella) for rt PCR did not include any IgM positive, I am not surprised to have negative signals in PCR. For this, the criteria for selection of samples should be revised and the results of the study reconsidered.

**Author’s response**:

We sincerely appreciate the reviewer's valuable insights regarding sample selection criteria and PCR testing. We fully acknowledge that PCR-negative results in IgM-negative samples are biologically plausible. We have thoroughly revised the method section to clear this issue and rephrased the text (Lines 296-298).

31) Lines 263-266: are "mucosal bleeding", "bloody vomit", or "epistaxis" most common symptoms in the classical clinical picture? Authors should check the frequency of these symptoms in common RVF infections.

**Author’s response**:

We sincerely appreciate the reviewer's insightful question regarding hemorrhagic manifestations in RVF. As detailed in Lines 303-305 and Table 4, none of the enrolled febrile patients reported a history of mucosal bleeding, hematemesis, or epistaxis within 30 days.

32) The titles of the tables are very long. Shorten!

**Author’s response**:

We thank the reviewer for this helpful suggestion. We have shortened all table titles while maintaining their completeness.

33) RVF and Coxiella results are not presented.

**Author’s response**:

We acknowledge that the initial presentation of the RVF and Coxiella results lacked clarity. We have thoroughly revised each section based on the newly added description and we believe the updated presentation communicates both sets of infections.

**Discussion**

34) Lines 302-303: would the fact of having IgM negative for both pathogens lead the authors to screen IgG positive samples through rt PCR? If yes, this should be pointed out somewhere in the methods to give a logical thread of testing in the study.

**Author’s response**:

We thank the reviewer for this valuable observation. As detailed in our responses to Questions 22, 29 and 30, we have reanalyzed all blood samples to check the presence of both pathogens’ genomic markers to:

- Exclude active infections during seroconversion windows
- Resolve methodological ambiguities

These testing algorithms are illustrated in (Fig 2; Lines 213-216; 296) and supported by an expanded discussion of their implications (Lines 341-345).

35) Lines 353-357: correctly number the points in the limitations section.

**Author’s response**:

We sincerely appreciate the reviewer’s attention to this concern. We have corrected the numbering of the limitations in the manuscript (Lines 389–398).

36) Diagnostic tools, cut-off for assays interpretation, the time elapsed between disease onset and care seeking, samples collection, combining point-of-care to clinical pictures in the ground conditions to quickly triage and isolated patients should be sufficiently discussed.

**Author’s response**:

Thank you for these overall comprehensive comments and suggestions. We agree that these critical methodological aspects, findings, and their practical implications warrant comprehensive discussion. Taking our primary objective into consideration, we have addressed these points across multiple manuscript sections. Key discussions include:

1. **Diagnostic tools and Cut-offs:** presented in Methods (Lines 166–208), including:

- ELISA antibodies interpretation for RVF virus (Lines 183-186; 192_ref; 231_ref; 240-241) and for *C. burnetii* (Lines 204_ref; 208_ref; 233_ref; 241-245),
- ELISA performance sensitivity/specificity (Lines 186-187; 192-193, 204-205, 209),
- RT-PCR Ct thresholds (≤36) (Lines 224-225),

2. **Timing of care-seeking and sample collection** impact on acute-phase detection discussed in:

- **Results**: Delayed presentation (mean: 9.5 days) (Line 303)
- **Discussion** (Lines 341–342, 366-367): Consequences for RVF/*C. burnetii* diagnosis
- Limitations (Lines 390-391): Potential underestimation of acute cases

3. **Point-of-Care (POC) integration:** While our study design (prevalence-focused) did not evaluate POC tools, we highlighted their potential importance in the Discussion section based on literature (Lines 347-349, 383-384).
37) From all changes, a conclusion can be re-written to include any change.

**Author’s response**:

We sincerely appreciate the reviewer’s constructive suggestion. We have carefully revised the conclusion section to incorporate changes made during the revision process, emphasize their public health significance, and articulate specific directions for future research.

**Reviewer #2**:

Well-written paper that addresses a need in this geographic region. Primary prevention efforts regarding Coxiella exposure could be further discussed but realize this is challenging in this setting due to resource limitations.

**Author’s response**:

We sincerely appreciate the reviewer's positive assessment of our work and valuable suggestions regarding Coxiella prevention. While we agree that primary prevention strategies (e.g., livestock vaccination, pastoral and agropastoral community zoonotic infection training) are critically important, their implementation remains challenging in our resource-limited setting. We have added recommendations (Lines 375-377) to highlight this key area for future intervention.

**References**

Sumaye, R., Jansen, F., Berkvens, D., De Baets, B., Geubels, E., Thiry, E. & Krit, M. 2019. Rift Valley fever: An open-source transmission dynamics simulation model. *PLoS One,* 14**,** e0209929.

Teunis, P. F., Schimmer, B., Notermans, D. W., Leenders, A. C., Wever, P. C., Kretzschmar, M. E. & Schneeberger, P. M. 2013. Time-course of antibody responses against Coxiella burnetii following acute Q fever. *Epidemiol Infect,* 141**,** 62-73.

van Wijk, M. J., Hogema, B. M., Maas, D. W. & Bokhorst, A. G. 2011. A Q Fever Outbreak in the Netherlands: Consequences for Tissue Banking. *Transfus Med Hemother,* 38**,** 357-364.
